# Skill Acquisition Methods Fostering Physical Literacy in Early-Physical Education (SAMPLE-PE): Rationale and Study Protocol for a Cluster Randomized Controlled Trial in 5–6-Year-Old Children From Deprived Areas of North West England

**DOI:** 10.3389/fpsyg.2020.01228

**Published:** 2020-06-17

**Authors:** James R. Rudd, Matteo Crotti, Katie Fitton-Davies, Laura O’Callaghan, Farid Bardid, Till Utesch, Simon Roberts, Lynne M. Boddy, Colum J. Cronin, Zoe Knowles, Jonathan Foulkes, Paula M. Watson, Caterina Pesce, Chris Button, David Revalds Lubans, Tim Buszard, Barbara Walsh, Lawrence Foweather

**Affiliations:** ^1^Research Institute for Sport and Exercise Sciences, Liverpool John Moores University, Liverpool, United Kingdom; ^2^Institute for Health and Sport, Footscray Park Campus, Victoria University, Melbourne, VIC, Australia; ^3^Physical Activity Exchange, Research Institute for Sport and Exercise Sciences, Liverpool John Moores University, Liverpool, United Kingdom; ^4^School of Education, University of Strathclyde, Glasgow, United Kingdom; ^5^Department of Movement and Sports Sciences, Ghent University, Ghent, Belgium; ^6^Institute for Sport and Exercise Sciences, University of Münster, Münster, Germany; ^7^Department of Movement, Human and Health Sciences, University of Rome “Foro Italico”, Rome, Italy; ^8^School of Physical Education, Sport and Exercise Sciences, University of Otago, Dunedin, New Zealand; ^9^Priority Research Centre in Physical Activity and Nutrition, School of Education, The University of Newcastle, Callaghan, NSW, Australia; ^10^School of Sport Leisure and Nutrition, Liverpool John Moores University, Liverpool, United Kingdom

**Keywords:** movement competence, low socioeconomic status, executive function, self-regulation, intervention, motor learning, pedagogy, mixed methods

## Abstract

**Background:**

There is a need for interdisciplinary research to better understand how pedagogical approaches in primary physical education (PE) can support the linked development of physical, cognitive and affective aspects of physical literacy and physical activity behaviors in young children living in deprived areas. The *Skill Acquisition Methods fostering Physical Literacy in Early-Physical Education (SAMPLE-PE)* study aims to examine the efficacy of two different pedagogies for PE, underpinned by theories of motor learning, to foster physical literacy.

**Methods:**

SAMPLE-PE will be evaluated through a cluster-randomized controlled trial targeting 5–6 year old children from schools located in areas of high deprivation in Merseyside, North-West England. Schools will be randomly allocated to one of three conditions: *Linear Pedagogy*, *Non-linear Pedagogy*, or Control. Non-linear and Linear Pedagogy intervention primary schools will receive a PE curriculum delivered by trained coaches over 15 weeks, while control schools will follow their usual practice. Data will be collected at baseline (T0), immediately post-intervention (T1), and 6 months after the intervention has finished (T2). Children’s movement competence is the primary outcome in this trial. Secondary outcomes include physical activity, perceived competence, motivation, executive functions, and self-regulation. An extensive process evaluation will also examine implementation factors such as intervention context, reach, dose, fidelity and acceptability.

**Discussion:**

The SAMPLE-PE project will enable better understanding surrounding how to operationalise physical literacy through enrichment of PE practices in early PE. The study will provide robust scientific evidence regarding the efficacy of underpinning PE pedagogy with theories of motor learning to promote the development of physical literacy.

**Trial Registration:**

Retrospectively registered on 5th September 2018 at ClinicalTrials.gov, a resource provided by the U.S. National Library of Medicine (Identifier: NCT03551366).

## Introduction

### Physical Literacy and Physical Education

Physical literacy can be understood as the embodied relationship between a child’s movement competence (physical), motivation and confidence (affective), knowledge and understanding (cognitive), and also their environment, which shapes movement and ongoing physical activity behaviors (Whitehead, 2010; Cairney et al., 2019). Across the globe, primary school PE curriculums, national standards and policies reference the support of the whole child, including physical, affective, cognitive and social development (The Australian Curriculum Assessment and Reporting Authority, 2012; Department for Education, 2013; United Nations Educational, Scientifc and Culture Organisation, 2015) thereby advocating the importance of physical literacy (Shape America, 2013; Sport England, 2013; Tremblay et al., 2018). It is widely accepted that early quality PE experiences are crucial for laying a strong foundation to support children on their physical literacy journey (Whitehead, 2010; Dudley, 2015). Nevertheless, there is a need for interdisciplinary research into physical literacy to better understand how pedagogical practices can foster physical literacy in early primary school.

### Supporting Physical Literacy Through Movement Competence

Although physical literacy is considered a holisitic concept with relevance through the life course, the early to middle childhood period is particularly important for nurturing the development and acquisition of foundational movement skills (e.g., running, jumping, catching, kicking) and abilities (e.g., agility, balance, coordination) (Whitehead, 2010; Giblin et al., 2014; Hulteen et al., 2018) collectively known as movement competence. Movement competence exists across a spectrum of human movement and is dependent upon an individual’s capacity to control, coordinate and perform movement skills efficiently (movement proficiency), as well as to adapt, attune and combine movement skills, creating novel functional solutions (movement creativity) across a broad range of physical activity and sporting contexts (Orth et al., 2017; Bardid and Utesch, 2018; Ng and Button, 2018). The ability to efficiently and functionally adapt, combine and execute movement skills requires emotional regulation, perceptual skills and a high degree of knowledge and understanding of the task at hand; the process of learning foundational movement skills will therefore drive the emergence of all aspects physical literacy in children (Rudd et al., 2016). Thus, supporting movement competence is considered central to fostering meaningful experiences in PE (Beni et al., 2017) therefore nurturing the physical literacy journey (Roberts et al., 2019b).

Low levels of movement competence have been reported among 4–8 year old primary school-aged children in western countries (Bardid et al., 2015; Foulkes et al., 2015; Morley et al., 2015). In particular, children from areas of relatively high deprivation in England (as calculated using the home postcode and information from domains including income, employment, education, health, crime, barriers to housing and services, as well as the living environment: see English indices of deprivation; Ministry of Housing Communities Local Government, 2018) have less developed movement skills than their peers from more affluent areas (Foulkes et al., 2015; Morley et al., 2015; Barnett et al., 2016). Children living in more deprived areas may require targeted movement competence interventions in PE due to a lack of opportunities to take part in physical activity outside of school or safe outdoor spaces within their community (Foulkes et al., 2015; Morley et al., 2015; Barnett et al., 2016). Low movement competency among more deprived children is a concern because children with low levels of movement competence have lower cardiorespiratory fitness, and are more likely to be overweight or obese, compared to children who perform these skills well (Lubans et al., 2010; D’ Hondt et al., 2014; McWhannell et al., 2018). From an affective perspective, children with high movement competence have been found to have higher perceived competence (Barnett et al., 2011; Liong et al., 2015; Duncan et al., 2018) which is important because children who feel confident whilst participating in PE are more likely to enjoy involvement, and consequently feel intrinsically motivated to continue effort and participation in all forms of physical activity. From a cognitive perspective, the ability to perform complex movement skills is positively associated with higher-order cognitive skills, i.e., core executive functions: working memory, inhibitory control and cognitive flexibility (Van Der Fels et al., 2015; Oppici et al., 2020), that allow children to manage their thoughts, actions and emotions in order to accomplish everyday tasks, and also to plan, organize and manage their time effectively. The development of complex movement skills through well-designed PE lessons can act as a ‘carrier’ of higher-order cognitive skill learning beyond those achieved through traditional classroom-based activities (Mavilidi et al., 2018). Behaviorally, children with higher levels of movement competence are more likely to be physically active during childhood, which in turn tracks into adolescence (Foweather et al., 2014; Holfelder and Schott, 2014; Lai et al., 2014; Cohen et al., 2015), determining positive trajectories of health (Robinson et al., 2015). In sum, poor movement coordination development among children living in areas of high deprivation may have wide-reaching adverse effects on their perceptual skills, cognition, social and emotional development and health (Leonard and Hill, 2014; Libertus and Hauf, 2017). Early intervention is seen as crucial given that an increasing proportion of young children have poor movement competence (Bardid et al., 2015; Foulkes et al., 2015; Morley et al., 2015). Whilst these articles highlight the potential benefits of movement competence, much of the research to date is cross-sectional or longitudinal (Holfelder and Schott, 2014; Robinson et al., 2015). There is a need for more experimental research within PE to provide robust evidence for movement competence influencing not only physical, but also cognitive and socio-emotional aspects of physical literacy (Whitehead, 2010; Dudley, 2015; Cairney et al., 2019).

### Use of Pedagogy in Movement Competence Interventions

In order for children living in deprived areas to develop high movement competence, it is important that they can access a PE curriculum with a strong theoretical basis, delivered by skilled practitioners, using systematic, progressive and developmentally-appropriate approaches to learning (Sweeting and Rink, 1999; European Physical Education, 2009). There have been a number of PE-based curriculum intervention studies which have focused on early primary school children’s development of foundational movement skills such as object-control (e.g., catching, throwing, kicking) and locomotor (e.g., running, hopping, jumping) skills (Robinson et al., 2015; Tompsett et al., 2017). While, in general, these interventions were successful, there is no clear indication in terms of the most effective pedagogy, curriculum, teaching behaviors and/or instructional strategies (Morgan et al., 2013; Robinson et al., 2015; Beni et al., 2017; Tompsett et al., 2017; Wick et al., 2017). Research in motor learning and control has advanced our knowledge about the physical, perceptual and cognitive processes involved in the learning of movement (Chow et al., 2016; Schmidt, 2019). These theoretical approaches can be used to inform the design of optimal learning environments to develop movement competence and support more broadly, physical literacy within primary school PE.

#### Linear Pedagogy

A popular pedagogical approach for teaching PE in young children is the Direct Instruction Model (Metzler, 2017). The main aim of this pedagogical model is to create ‘closed’ environments that are highly structured, and overly constrained environments that first develop content (i.e., ‘technical proficiency’) before being applied to various contexts (i.e., within the ‘open’ environment of a game or performance setting) (Blomqvist et al., 2001; Kirk, 2010). This pedagogical model aligns with cognitive and linear approaches to motor learning in accordance with Information Processing Theory (Kirk et al., 2006; Ennis, 2017; Schmidt, 2019). Lesson design structure and teaching methods hold with the premise that learning (movement) is a gradual linear process where the development of a skill progresses through main observable stages of learning (cognitive, associative, and autonomous) characterized by a reduction in cognitive processing when performing the movement skill (Fitts and Posner, 1973). This linear pedagogy includes both prescriptive (e.g., following technical demonstrations and instructions from the teacher) and repetitive actions (e.g., repetition targeting the replication of the optimal technique), where variability is reduced until a performer can execute a movement skill efficiently and reliably (Schmidt, 2019). Feedback is largely a one-way process from the teacher to the child for error detection and correction.

To fully appreciate the potential of these linear pedagogical curriculums to foster physical literacy in children, it is important to consider the individual learning experience. Children’s perceptions of competence and motivation may be influenced through emphasis and development of movement proficiency in one optimal technique and may result in a sense of mastery over the skill, leading to early feelings of success that should increase perceptions of competence, contributing to higher levels of motivation in the lesson (Ryan and Deci, 2000, 2017). From a cognitive perspective, it is suggested that pedagogies that follow a linear progression of skill learning may support the natural scaffolding of executive functions as inhibitory control and working memory, providing the architecture for cognitive flexibility to be built upon (Deák and Wiseheart, 2015; Van Der Fels et al., 2015; Pesce et al., 2016a). This is due to the learning design of Linear Pedagogy first constraining children to practice skills in isolated environments before moving into a game or performance situation that will require cognitive flexibility. Evidence suggests that PE interventions aligned to the Direct Instruction Model and/or reflecting linear methods of skill learning are an effective teaching strategy for supporting young children to develop movement skill proficiency (Morgan et al., 2013; Tompsett et al., 2017; Wick et al., 2017). However, some of this evidence can be interpreted as low-quality, while many studies lack long-term follow-up (Morgan et al., 2013; Wick et al., 2017). Further, while studies have documented increases in movemennt skill proficiency, there is a lack of evidence for movement creativity outcomes, and limited evidence of concomitant increases in affective and cognitive domains, as well as physical activity behavior (Morgan et al., 2013; Lai et al., 2014; Tompsett et al., 2017).

#### Non-linear Pedagogy

The theory of Ecological Dynamics, offers a Non-linear perspective on the learning and development of movement skill (Chow et al., 2007). According to Ecological Dynamics, goal-directed movements are the product of the interaction between personal, environmental and task constraints (Chow et al., 2011). From this perspective, motor learning is not simply a matter of processing information and accruing representations (as is the case in cognitive theories) (Bailey and Pickard, 2010). Learners are regarded as complex adaptive systems who are presented with opportunities for action (affordances) from their environment. The concept of affordances highlights the interaction between the environmental features and functional capabilities of the individual child. Children are able to identify affordances within their environment based on their level of movement development (Newell, 1986). Newell (1986) also proposed three observable stages of learning coordination, control and skill. At an unconscious level, the learner is solving the degrees of freedom problem in early skill learning through freezing out or locking joints and body segments, allowing them to achieve the movement goal in a rudimentary form. As they move through the stages of learning will see an unlocking of degrees of freedom eventually in the skill stage learners are able to exploit environmental features to enhance and execute goal-directed movements in an energy-efficient manner that appears almost effortless. In Non-linear Pedagogy, the teacher’s role is to design learning experiences in which the child’s capability and environmental opportunities are closely aligned, creating opportunities for goal-directed movement (i.e., affordances). One way for the teacher to create affordances and channel the child’s movement competence development is through manipulation of task and environmental constraints (e.g., rules, space, equipment). This manipulation aims to promote an external focus of attention that limits the allocation of resources to motor coordination and control processes and facilitates the implicit learning of movement skills (Profeta and Turvey, 2018). The child is left free to experiment by performing, adapting and creating movement solutions that best answer their individual needs within a given context. Traits of non-linear pedagogy can be observed in pedagogical models such as ‘Teaching Games for Understanding’ and teaching styles such as inquiry-led, co-operative, and discovery learning (Mosston, 2002; Tan et al., 2012; Metzler, 2017).

A Non-linear pedagogical approach to learning in PE also has implications for a child’s affective and cognitive development, and physical activity behavior. Similar to linear pedagogies, the development of movement competence may increase perceptions of competence, contributing to generally higher levels of motivation. Moreover, Non-linear Pedagogy may have specific implications for children’s autonomous motivation for PE, as children are provided with choice and freedom to move in different ways within their PE lessons, which could enhance their enjoyment and perceptions of autonomy (Ryan and Deci, 2000, 2017). Further, the focus on finding different movement solutions to achieve a goal may see a shift in how the child views competence, away from an ‘ideal’ movement performance toward functional, creative movements (Lee et al., 2014; Moy et al., 2016). The respect the teacher or coach gives to the child’s ability to explore, learn, work with others and problem solve may also enhance the child’s feelings of relatedness (Ryan and Deci, 2000, 2017). A Non-linear Pedagogy may have a favorable impact on the development of executive functions as the search for different solutions of an emerging movement problem involves inhibiting routines, working with ideas in working memory and flexibly shifting between potential solutions (Pesce et al., 2016b) and the non-linear instructional environments designed by supportive instructors can elicit children’s commitment and emotional investment (Diamond and Ling, 2019). From a behavioral perspective, it is suggested that the long-term effect of this pedagogy is that children could acquire a wide range of functional movement solutions that are both adaptable and attuned across a variety of physical activity environments (Renshaw et al., 2010; Chow and Atencio, 2014).

While the potential holistic benefits of Non-linear Pedagogy for primary school PE have been argued (Renshaw et al., 2010; Chow and Atencio, 2014) and discussed with reference to physical literacy (Roberts et al., 2019a) to date there is limited evidence of the utilization of Non-linear Pedagogy in primary PE and little empirical evidence in support of these claims (Tompsett et al., 2017). Some PE interventions with characteristics of Non-linear Pedagogy have targeted and demonstrated improvements in movement competence among primary school children, relative to control conditions following usual PE practice (Miller et al., 2016; Pesce et al., 2016a). Miller et al. (2016) also demonstrated increased pedometer steps (physical activity behavior) in PE following the intervention; Pesce et al. (2016b) reported that movement competence (object control skills) outcomes mediated executive function (inhibitory control) outcomes. However, the observed benefits did not extend from actual movement competence to perceived athletic skill competence (Miller et al., 2016), or from inhibition to other core executive functions (Pesce et al., 2016a). Thus, further research is required to demonstrate the efficacy of Non-linear Pedagogy in PE to promote the development of movement competence and the generalizability of outcomes to aspects of physical literacy beyond the physical domain.

### Aims of the Current Study

The purpose of the SAMPLE-PE study is therefore to assess the efficacy of utilizing *Linear* and *Non-linear* pedagogy within PE to promote movement competence (proficiency and creativity) and wider cognitive and affective aspects of physical literacy in 5–6 year old children from deprived areas of North West England. The SAMPLE-PE intervention is focused on PE as an ideal setting to reach all children. SAMPLE-PE targets the early primary school PE curriculum as this is the first formal opportunity for children to participate in PE in England and young children from deprived areas in a major city in north-west England have been found to be in the greatest need of such an intervention (Foulkes et al., 2015). Specifically, the main objectives of the study are to assess the efficacy of PE pedagogies (*Linear* or *Non-linear*) delivered over 15 weeks, compared to standard PE practice, on 5- and 6- year-old children’s movement competence (physical domain), perceived movement competence and self-determined motivation (affective), executive function (cognitive), self-regulation (cognitive-affective), and physical activity (behavioral). A further objective of the study is to explore the potential mediating mechanisms for any intervention effects, and in particular whether increases in movement competence mediate differential effects of Linear and Non-linear Pedagogy across other elements of physical literacy. The joint focus on executive function and self-regulation is targeted to couple the more common view on the movement competence-cognition relationship, mainly focused on ‘cool’ executive functions elicited under affectively neutral conditions (Van Der Fels et al., 2015) with a still under-considered view on ‘hot’ executive function processes performed in affectively salient contexts (Zelazo and Carlson, 2012), as those involved in self-regulation (Lakes and Hoyt, 2004; Zelazo and Carlson, 2012; Lakes et al., 2013).

### Hypotheses

Based on previous literature (Morgan et al., 2013; Tompsett et al., 2017), we expect that children who participate in the *Linear* and *Non-linear* Pedagogy interventions will demonstrate greater improvements in movement competence compared to children following standard PE practice. It is also expected that children in the Non-linear Pedagogy intervention will demonstrate greater movement creativity but lower technical movement proficiency than children in the Linear Pedagogy group (Lee et al., 2014). Furthermore, children in Linear and Non-linear Pedagogy interventions will show greater gains across physical literacy elements (affective [perceived competence and motivation], cognitive [cool executive functions], cognitive-affective [self-regulation] and behavioral [physical activity]) than children in standard PE practice. Finally, it is also expected that the Non-linear Pedagogy intervention will see greater improvements in children’s affective (motivation), cognitive (cool executive functions: cognitive flexibility, working memory, and inhibitory control), cognitive-affective (self-regulation) domains than the Linear Pedagogy intervention (Lee et al., 2014; Alvarez-Bueno et al., 2017; Vazou et al., 2019).

## Methods

### Design

**FIGURE 1:**
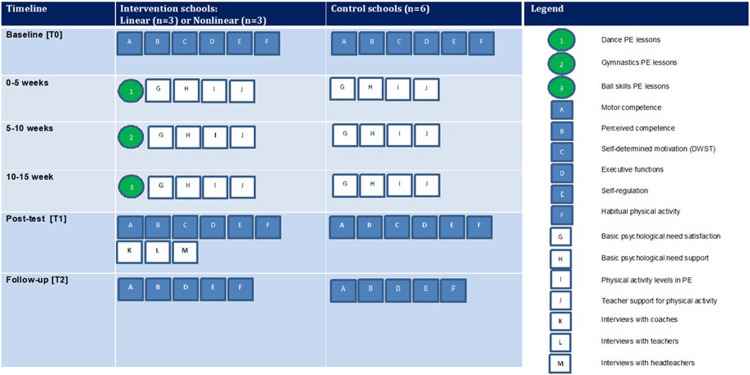
Schematic overview of SAMPLE-PE study design and evaluation components.

**FIGURE 2:**
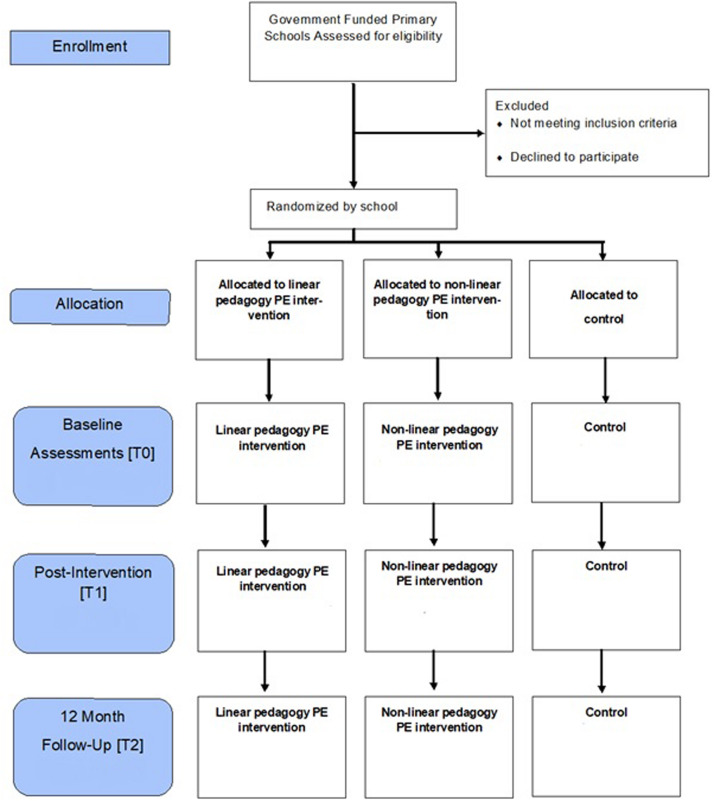
SPIRIT participant timeline.

A cluster-RCT will be conducted to evaluate the efficacy of the SAMPLE-PE pedagogy interventions that aim to improve movement competence and other key aspects of children’s holistic development in year 1 children (5–6 years) in 12 government-funded primary schools. The trial has received institutional research ethics committee approval (Reference 17/SPS/031), and is registered (ClinicalTrials.gov identifier: NCT03551366). A schematic overview of the intervention and evaluation components is shown in Figure 1, while the flow diagram of schools through the study is shown in Figure 2. The UK school academic calendar spans September to the middle of July. Data collection will occur over 14 months with measurements at baseline (T0, January–February, 2018) and post-intervention (T1, June–July, 2018), whilst children are in year 1 of primary school, with a follow-up planned for 6 months after the intervention has finished (T2, January–February, 2019; year 2 of primary school; 1 year post-baseline assessments). The design, conduct and reporting of this cluster RCT will adhere to the Consolidated Standards of Reporting Trials (CONSORT) guidelines for group trials (Schulz et al., 2020) and the Standard Protocol Items: Recommendations for Interventions (SPIRIT) checklist (Supplementary File 4) (Chan et al., 2013).

### Sample Size and Statistical Power

Based on previous studies (Morgan et al., 2013), we anticipate a small to medium effect size of *d* = 0.4 for changes in movement competence. In accordance with CONSORT guidelines (Schulz et al., 2020) our power calculations were adjusted for the clustering of effects at the class level. Adjusting for clustering at class level, we used a correction factor of [1+(m-1) × ICC], with participants m per class and the intraclass correlation ICC coefficient. Assuming an average class size of about 20 participants and an ICC for movement competence of 0.16 [based on TGMD-2 data of 8 classes from 7 to 8 year-olds (Rudd et al., 2016), the correction factor is 4.04 (i.e., 1+(20-1) × 0.16) (Lubans et al., 2016)]. The power calculation to detect within-between interactions for three groups and across three time points with 90% power, α levels set at *p* < 0.05 and *r* = 0.5 suggested a minimal sample size of 54 children. The final power calculation including the correction factor indicated sample size of 218 children. Allowing for 20% dropout at each time points (Foulkes et al., 2017), the aim of this study will be to have a sample of at least 314 children.

### Settings and Participants

Eligible government-funded primary schools located within a large city in North West England will be invited to participate in the study via email and telephone. Eligible schools are required to be located within an area ranked within the most deprived tertile for the English population, as measured by the 2015 English indices of deprivation index (Ministry of Housing Communities Local Government, 2018). Representatives from eligible schools will subsequently be invited to an information meeting with the research team, where they will be given an in-depth overview of the project. Signed consent will be obtained from headteachers for recruitment, data collection and potential delivery of PE by the research team. Eligible children from year 1 classes will then be invited to participate in the study via a parent/carer and child invitation pack, including information sheets, consent forms, parent and child characteristics questionnaire, child medical information form, and child assent form. Children that are not able to participate in PE (e.g., due to medical conditions) or those with profound learning disabilities and formally recognized special educational needs (e.g., behavioral issues, speech and language impairment) will be excluded from assessments and data analysis. Children that do not return parent consent forms will be exempt from the research, but able to participate in PE lessons.

### Blinding and Randomization

For practical reasons, it will not be possible to blind the researchers, teachers, and coaches to group allocation. Following collection of headteacher consent, randomization will take place at the school (cluster) level. Schools will then be matched based on the number of students enrolled and level of deprivation identified using the school postcode (Ministry of Housing Communities Local Government, 2018). Following this, schools will be randomly allocated to an intervention condition or control group using a computer-based random number producing algorithm by an independent researcher not associated with the study. This method ensures that schools had an equal chance of allocation to each group.

### Intervention

#### Overview

SAMPLE-PE aims to explore the efficacy of two PE pedagogies (Non-linear Pedagogy and Linear Pedagogy), delivered through a 15-week PE curriculum in primary schools situated in areas of high deprivation. Each school being assigned to one of three conditions: Non-linear Pedagogy PE intervention, Linear Pedagogy PE intervention or control group (standard PE curriculum). All groups will have the same dose of PE (i.e., 2 × 60 min weekly PE lessons, for 15 weeks).

The SAMPLE-PE intervention curriculum for both the Linear Pedagogy and Non-linear Pedagogy arms will consists of 3, 5-week phases of lesson delivery commencing 2 weeks after baseline assessments. The first phase focuses on dance, the second on gymnastics and the final phase on ball sports. Each phase has its own scheme of work, which includes five lesson objectives, each taught over a two lesson period, and delivered in school during existing PE curriculum time. The lesson objectives are aligned to the aims of the English national curriculum (Department for Education, 2013) and are identical in both Linear and Non-linear Pedagogy schemes of work, but the content was differentiated by pedagogical approach in an effort to support the development of the lesson plans (described in detail below). Lessons will be delivered by trained coaches, with 45 min of on-task teaching time of the total 60 min overall lesson time, culminating in a total of 30 PE lessons.

#### Training Coaches for Intervention Delivery

The present study is both an efficacy and an effectiveness trial. Given that there is evidence that some generalist primary school teachers lack the confidence and competence to effectively teach PE (Morgan and Hansen, 2008), coaches will be recruited to deliver the Linear and Non-linear Pedagogy PE interventions. This approach also corresponds with current practice in primary PE in England, as the majority of primary schools currently source external providers who employ sports coaches to deliver PE (Griggs, 2016). Sport coaches will be recruited through advertisements aimed at postgraduate and undergraduate students undertaking Sports Coaching or PE courses or via the university’s in-house sports coaching provider. Applicants will be shortlisted if they have a level 2 coaching qualification in any sport, meaning that they can independently plan, prepare and deliver sessions and they have basic emergency first aid, safeguarding and protecting children certificates. Further, it is desirable that coaches will have at least 1-year’s coaching and/or PE teaching experience in a primary school or sports club setting. Recruited coaches will then be invited to attend a bespoke 5-week training programme. This training aims to develop the coaches’ knowledge and skills to deliver either a Linear (operationally through Direct Instruction Model) or Non-linear Pedagogy SAMPLE-PE curriculum.

Prior to the start of the training programme, coaches will be asked to design and deliver a coaching session to year 1 children, which will be video recorded by the research team. The video recordings of the session will subsequently be analyzed by two members of the research team with expertise in both pedagogical approaches. This exercise will enable the research team to determine whether each coach’s style of delivery is consistent with direct instruction-based teaching characteristics of Linear Pedagogy or more consistent with inquiry-based and problem-solving teaching characteristics of Non-linear Pedagogy. Coaches will then be allocated to either a Linear or Non-linear 5-week pedagogy training programme based upon their observed teaching style. This programme will comprise 3 h training each week delivered by the research team within a local primary school. Each training session will include a 90-min classroom theory session on either Linear or Non-linear Pedagogy, with pedagogical content knowledge relating to dance, gymnastics and ball sports, and a 90-min practical session of PE delivery to year 1 and 2 primary school children. The practical sessions will consist of a 45-min model lesson delivered in the pedagogical style by a member of the research team who has recognized expertise in PE teaching (Roberts et al., 2019b) followed by the coaches implementing their own lessons in accordance with the respective pedagogy.

All coaches will be provided with a scheme of work, lesson plans and a pedagogical framework for each PE subject (dance, gymnastics, and ball sports), a resource pack covering key elements of their respective pedagogical approach and copies of recorded theory and practical lessons were put online as coaches’ resources. A key aspect of the coaches training is the DIFFerentiation framework (see Table 1). Coaches will be trained on how they should utilize powerful teaching strategies of demonstration, instruction, and feedback in line with their respective pedagogies (linear or non-linear). This framework was based upon research from either a cognitive approach or ecological approach to motor learning. Coaches will be asked to complete a self-reflection either via diary or audio recording (Gibbs, 1988) each week concerning their implementation of the respective SAMPLE-PE pedagogy principles. This self-reflection will form the basis of discussions in weekly meetings with a member of the research team, alongside any changes necessary to the next week’s lesson plans. Coaches will also have the opportunity to access telephone support and a critical friend from the research team throughout the intervention delivery schedule.

**TABLE 1:** DIFFerentiation framework used to support coaches teaching behaviors in the linear and non-linear pedagogy SAMPLE-PE curriculums.

	Linear pedagogy			Non-linear pedagogy	
	
General assumptions (‘DIFFerentitaion’)	Children in the autonomous and associative stage of learning	Children in the cognitive stage of learning	General assumptions (‘DIFFerentitaion’)	High motor competence children	Low motor competence children
	**(Fitts and Posner, 1973)**			**(Newell, 1986)**	

**Demonstration Isolated demonstrations of a motor skill by an adult or competent child is to be promoted as it offers a unique opportunity for learners to gather information about appropriate coordination patterns which could benefit performance.** (Shea et al., 1999)	Demonstration provided after practice of a task lead to stronger retention of learning than demonstration prior practice (Blandin et al., 1999)	Demonstration of a skill by an individual presenting high proficiency is beneficial for motor learning. (Blandin et al., 1999)	**Demonstration Adult demonstration is avoided as NLP encourages more than one optimal way to move in a functional manner.** (Williams and Hodges, 2005; Chow et al., 2016)	No demonstration is given as NLP suggests that it is more or less redundant as they are at the level where further demonstration will no longer provide them with useful information. (Chow et al., 2016)	A few highly competent children to demonstrate the movement in context so that the observing moderate to low competent children can see what they could do within their own movement. (Chow et al., 2016)

**Instruction The use of instruction should have both an internal (skill focus) and external focus of attention is allowed.** (Beilock et al., 2002; Wulf, 2007)	Verbal instructions should focus on movement outcomes rather than on the movements required by the task. (Beilock et al., 2002)	A skill focus instruction is encourage to support early acquisition of the skill as it has been found to be more effective in skill execution. (Beilock et al., 2002)	**Instruction The use of instruction is not encouraged if it is needed it should be short and not be prescriptive. Instead coaches were encouraged create games, scenarios and to manipulate task constraints to promote skills being learnt implicitly.**	Use of questioning and external focus as it allows children to problem solve toward a movement solution. (Chow et al., 2016) Coach use STEP framework to manipulate task constraints	If the child has no previous experience of the motor skill, the use of analogies can help as it chunks a large amount of information together that frees up mental capacity providing an external focus of attention. (Chow et al., 2016)

**Feedback and Frequency Feedback is a powerful tool in the coaches toolbox and should be used at the coaches discretion based on their judgment of a child’s motor competence. Feedback can either take the shape of knowledge of results or knowledge of performance.** (Sherwood, 1988; Sullivan et al., 2008)	Feedback should be provided only when error are large enough to warrant attention. (Sherwood, 1988)	Providing verbal feedback after each trial or as much as possible during early stages of acquisition is a priority (Sullivan et al., 2008) Practitioner should identify the component of the skills that needs to be learned, determine which is most critical for learning and prioritize feedback about the critical component of the task though this should not happen after every trial. (Weeks and Kordus, 1998)	**Feedback and Frequency Feedback should focus on children finding different movement solutions. Feedback is kept to a minimum and only used when children get stuck or to create instability in movement pattern.**	External feedback should only be given if they miss the mark. If they achieve the desired outcome, feedback is not necessary (Hodges and Franks, 2001)	Feedback should never be corrective. The coaches feedback should be minimal and if used should promote an external focus of attention. As with instructions analogies can be useful to support learning. Coaches can also utilize STEP framework to manipulate task constraints (Chow et al., 2016)

#### Linear Pedagogy

The SAMPLE-PE Linear Pedagogy intervention postulates that movement learning is a process that unfolds in identifiable linear phases (Schmidt, 2019). The Direct Instruction Model pedagogical approach will be used by coaches to create a PE environment where the learner first replicates the coaches’ technique, as well as scaffolding activities; starting with low environmental variability, as skill improves the learner will be placed into incrementally more variable and dynamic environments. To support the coaches’ learning design and delivery, they were trained to utilize three models: Fitts and Posner’s stages of learning (Fitts and Posner, 1973), Gentile’s taxonomy (Gentile, 2000) of movement skills, and the challenge point framework (Guadagnoli and Lee, 2004).

Coaches will be trained to identify children in each of Fitts’s and Posner’s three stages of learning (cognitive, associative or autonomous) and then, prior to the start of the PE lesson, to use this knowledge to modify lesson activities using Gentile’s taxonomy. The 16 categories of the taxonomy lead the coach through a logical sequence of potential progressions and force the coach to consider two main perspectives: the environmental context in which the skill takes place and the function that the movement skill must fulfill. Using Gentile’s taxonomy, a coach can manipulate the skill to its simplest form, in which the child has a stable base without any object manipulation and in an environment free from distraction. If the coach believes that a child or class of children have higher competence, they can use Gentilie’s taxonomy to create a skill context that is far more challenging, i.e., body in motion, manipulation of an object, and environmental factors dictating movement skill responses (Gentile, 2000). To support children’s individual needs during the lesson, coaches utilize the challenge point framework (Guadagnoli and Lee, 2004), which indicates that there is an optimal level of challenge for children to maximize learning in a given activity. Each lesson activity represents different challenges for children at different stages of learning a movement skill. The level of difficulty will be dependent upon a number of key variables: the skill level of the performer, the complexity of the activity, and the environment in which the activity is taking place. The more difficult the activity, the greater the learning potential, though this is related to an increase in task difficulty, and as such, the performance of the learner is expected to decrease. Thus, learning is maximized in PE when a child is optimally challenged. This framework supports coaches to critically assess if learning is taking place and consider how they can support a child to maximize learning.

The Linear Pedagogy curriculum was guided by four principles:

(1) There is a correct optimal movement pattern for each foundational movement skill. This is based on the idea that is there is a movement trace that acts as a reference of correctness to guide a child’s movement. The coach therefore relies heavily on demonstrations of an optimal movement pattern as this offers a unique opportunity for learners to gather information about appropriate coordination patterns and task requirements which can benefit performance (Sweeting and Rink, 1999; Hayes et al., 2008).
(2) Movement skills are broken down or simplified into key components of a skill for learning, as performing an optimal movement pattern is often beyond the reach of children who are in the early stage of learning a skill.
(3) Movement variability is viewed as noise in the system, which the child has to reduce in their quest toward mastery of a skill. The coach overcomes this by repetitive practice of the skills, which gradually reduces the amount of variability in the system, and the result is an efficient, reliable and accurate movement skill performance.
(4) The focus of attention when performing a movement skill. The majority of research in this area highlights that promotion of an ‘external focus’ generally results in more effective performance and learning of a movement skill (Wulf, 2013). However, individuals in the cognitive phase of movement skill learning have been found to benefit from an internal focus of attention, e.g., a focus on the foot contact if dribbling a football (Beilock et al., 2002). Therefore, the SAMPLE-PE Linear Pedagogy curriculum coaches will be trained to create an internal focus of attention for children identified as in the cognitive phase of skill development (i.e., children with low movement competence), while for children progressing beyond this stage (i.e., children with higher movement competence), coaches focused on an external attention of focus.

The Linear Pedagogy PE curriculum was successfully trialed with year 1 children across three primary schools in summer 2016. A copy of the lesson plan can be found in Supplementary File 1.

#### Non-linear Pedagogy

Ecological dynamics considers individuals (or at a higher level of analysis, a class of children) to be complex and adaptive systems (Davids et al., 2012). If this theoretical premise is accepted there is, from a learning design perspective, considerable uncertainty as to how any particular PE lesson will unfold, and consequently lesson plans should act as a guide, rather than being adhered too strictly at the cost of learning opportunities. Coaches therefore need to adopt a frontloaded approach, whereby they consider in advance how any changes within the PE lesson may alter the learning of each child. While this may seem like an impossible task, there are some consistent variables across schools (e.g., class sizes, lesson duration, national PE curriculum objectives). Moreover, within the classroom there will be common constraints acting upon children such as their age, socio-economic demographic, and the school environment, which either facilitate or hinder motor learning. The research team and coaches will work together to identify common constraints for year 1 children, creating an expected range of variation that the coach could plan for and exploit during their PE lessons, allowing them to design more individualized and meaningful movement experiences for their children. It is important to highlight that this approach recognizes that it is impossible to repeat a movement identically from one attempt to the next (Newell, 1986). Thus, accepting variability in movement is central and the coaches’ role is to encourage participants to adapt their movements and continue to improve their technique.

In order to help the coaches deliver the Non-linear Pedagogy curriculum, they will be trained to utilize two models: Newell’s (Newell, 1986) model of motor learning, and the Space, Task, Equipment and People (STEP) framework (Youth Sport Trust, 2018). Newell (1986) model of motor learning is based on Ecological Dynamics and will be used to teach coaches that high movement competence is represented by a child’s ability to be creative and adaptable whilst succeeding in their performance of movement skills. Coaches will be trained to identify if children’s movement behaviors are in the coordination, control or skill stage of learning, and subsequently individualize the PE activity toward a child’s particular level of competence by changing one or more task constraints. The STEP framework (Youth Sport Trust, 2018) will support the manipulation of task constraints by increasing or reducing the likelihood of affordances, with the aim of enabling children to effectively solve movement problems.

Alongside these models, the Non-linear Pedagogy curriculum is underpinned by five core pedagogical principles:

(1) A representative learning design. Arguably, a common representative learning design for young children within a PE setting is fun (Headrick et al., 2015; Beni et al., 2017; Foweather and Rudd, 2020). Representative learning design also highlights the importance of skill transfer between multiple settings. For this to occur, it is important that there is a behavioral correspondence between learning and the child’s other performance environments, such as the playground, afterschool clubs and sport clubs.
(2) Movement-perception coupling must be maintained when performing skills. This means that skills are practiced in their entirety rather than broken down into component parts or in decontextualized fashion. Movement-perception coupling is seen as a micro (skill level) equivalent of the macro (environment) representative learning design. From a macro perspective, the movement-perception coupling is maintained, for example, within gymnastics lessons by having all equipment present throughout the duration of each lesson, improving their ability to self-regulate their behavior. At the level of the microstructure of practice, the coach does not prescribe the type of movement skill that the child should learn. Instead, the coach promotes creativity and exploration through the use of scenarios and/or mini-games that encourage children to explore and experiment with a broad range of movement skills, meaning movements are learnt in context, and the coach does not isolate skills or develop them by separating into components. Alongside this the coach employs the use of analogies and open-ended questions in the effort to encourage problem solving from the child rather than telling the child exactly what to do.
(3) An external focus of attention is considered necessary to support the acquisition of both creative and functional movement skills. Profeta and Turvey (2018) suggest that if the learner allocates attentional resources to the task and environment rather than to the own movements, movement coordination and control is delegated to the lower levels of the central nervous system where movement is less conscious and learning occurs implicitly. An external focus of attention allows for self-organization of movement patterns to meet the goal of the task, whilst an internal focus of attention promotes a conscious process which is believed to lead to an undesirable breakdown of movements (Wulf, 2007; Chow et al., 2016). To develop functional and adaptive movements, coaches were trained to create mini-games within the lessons, and to utilitize and build upon teaching methods such as analogies and questions. These type of activities create an external focus of attention.
(4) The application of constraints – boundaries or features that encourage the development of movement competence. There are three types of constraint: individual, environmental, and task (Pesce et al., 2016b). The coaches are able to make decisions on what task constraints to manipulate based upon their observations of children’s interactions with their environment and using their knowledge of Newell’s stages of learning and the STEP framework (Newell, 1986; Youth Sport Trust, 2018). For example, the coach could reduce or increase the playing Space, alter the rules of the Task, use different sized Equipment and/or change the number of People playing the game.
(5) Infusing perturbations within the learning process. This means that if the coach observes a child demonstrating a stable and functional movement skill, the coach will act to destabilize the skill by altering task constraints or changing the task goal. Changing task constraints will result in new affordances. It is important that the coach understands that it is acceptable for different children to display different movement solutions to the same task and that regression in skill is inevitable when altering constraints (such as equipment). As long as the skill is functional and achieves the outcome of the lesson, then it is to be accepted as a pertinent solution.

The Non-linear Pedagogy PE curriculum was successfully trialed with year 1 children across three primary schools in summer 2016. A copy of the lesson plan can be found in Supplementary File 2.

#### Control (*n* = 6 Schools)

Control schools will be asked to continue with their usual PE curriculum provision, and timetable and deliver 2 × 60 min PE lessons per week for 15 weeks. The control schools follow current national curriculum aims for PE in Key Stage 1 (early primary), which state that: *‘Pupils should develop fundamental movement skills, become increasingly competent and confident and access a broad range of opportunities to extend their agility, balance and coordination, individually and with others. They should be able to engage in competitive (both against self and against others) and co-operative physical activities, in a range of increasingly challenging situations’* (Department for Education, 2013). Information pertaining to the PE curriculum being delivered in control schools will be collected as part of a process evaluation (described later in secondary outcomes).

### Outcomes

Trained research assistants will undertake 2–3 days of data collection at participating schools across three time-points (see Figure 1). Demographic characteristics including child’s age, gender, ethnicity, and home postcode (used to classify children into deciles of deprivation level using the English indices of deprivation: Ministry of Housing Communities Local Government, 2018) will be collected at baseline through parent consent forms. A number of primary and secondary outcomes are measured through the study.

#### Primary Outcome

##### Movement competence

Movement competence will be assessed through a battery of assessments to examine both technical movement proficiency and movement creativity across different domains (locomotor, object-control, and stability skills). All movement competence assessments will take place during school hours within the school hall or playground and video-recorded for later analysis. Trained research assistants who have established acceptable agreement (80%) in terms of intra-rater and inter-rater reliability with pre-coded videos, will complete analysis of video recordings.

Movement proficiency (technique) will be assessed using the Test of Gross Motor Development-3 (TGMD-3; Maeng et al., 2016) and the Test of Stability Skills (Rudd et al., 2015). Specifically, six locomotor (run, gallop, hop, skip, horizontal jump, slide) and seven object-control (two-hand strike, one-hand strike, one-hand dribble, two-hand catch, kick, overhand throw, underhand throw) skills will be assessed using the TGMD-3 (∼30 min to complete). Proficiency at stability skills will be assessed using the three tasks (log roll, rock, back support) from within the Test of Stability Skills (∼15 min to complete). The psychometric quality of these assessments has been well-established (Rudd et al., 2015; Maeng et al., 2016). Participants will receive a verbal explanation and single demonstration from the assessor and are then given one practice attempt before undertaking two trials of each skill.

Movement creativity will be assessed using the Divergent Movement Ability Assessment (Cleland, 1990), which requires children to complete three stations, a stability skill station, a locomotor skill station and object control skill station (∼15 min to complete). In the stability station, children are asked to make as many shapes on or around the bench as they can. In the locomotor station, children are challenged to find as many different ways to move around the obstacle course as possible. Finally, in the object-control skill station, children will be asked to play with a large ball in a designated area, showing all the different skills and ways that they can play with the ball. For every station, children will complete two 90 s trials, during which, every 30 s the child will get a predefined prompt from the research assistant to support and encourage the child.

#### Secondary Outcomes

##### Physical activity

Participants will be asked to wear a monitor (accelerometer; ActiGraph GT9X, ActiGraph, Pensacola, FL, United States) on their non-dominant wrist continuously for 7 days to measure physical activity at each time point. Participants will be asked to wear their monitors at all times, and to remove them only for water-based activities. Accelerometers will be initialised at a sampling frequency of 30 Hz. During the monitoring period, children’s parents are asked to keep a diary in order to record any times when the monitor is taken off, any activities completed whilst the monitor is removed (e.g., swimming, bathing), and the time the monitor is put back on. A member of the research team will return to the school at the end of the 7-day period to collect the monitors and diaries. Accelerometry data will be used to examine within school, leisure (after-school and weekend), and habitual (total) physical activity levels. Children will be included in the analyses if they have worn the monitor for at least 10 h per day over 3 days, including one weekend day. Time spent in sedentary, light, moderate and vigorous activity will be determined using age- and- population-specific raw acceleration cut-points for the wrist-worn ActiGraph, developed through an ongoing research study (Crotti et al., 2020).

##### Perceived competence

Perceived physical competence (higher order construct) will be assessed using the corresponding subscale within The Pictorial Scale of Perceived Competence and Social Acceptance for Young Children (Harter and Pike, 1984). The Physical Competence subscale includes items 3, 7, 11, 15, 19, and 23 from the overall Pictorial Scale, and takes approximately 3 min to complete. Each item is scored on a four-point scale, where 4 represents the highest degree of perceived competence. The subscale score is computed by adding values of child responses and ranges from 6 to 24.

Perceived Skill Competence (lower order construct) will be assessed by the Pictorial Scale of Perceived Movement Skill Competence for Young Children 3rd Edition (Harter, 1978; Barnett et al., 2015). The Scale consists of 13 items with two subscales of six and seven items for “Locomotor Skill Perceived Competence” and “Object-Control Skill Perceived Competence,” respectively. Each item is scored on a four-point scale, where 4 represents the highest degree of perceived competence. Subscale scores are computed by adding values of child responses and range from 6 to 24 for locomotor and 7 to 28 for object control (higher values indicate higher perceived competence). All 13 items are summed to generate the Perceived Movement Skill Competence scale score, which ranges from 13 to 52 (higher values indicate higher perceived competence). The Pictorial Scale of Perceived Movement Skill Competence for Young Children is a valid and reliable instrument to assess perceived movement competence in young children (Barnett et al., 2015), taking around 5–7 min to complete.

##### Motivation and psychological needs satisfaction

Self-determined motivation and psychological needs satisfaction are difficult to assess in young children as traditional self-report measures are not appropriate (Sebire et al., 2013). Therefore, following Noonan et al. (2016) and Parker et al. (2018), we have developed a child friendly and age-appropriate Physical Education Motivation Assessment Tool (MAT-PE) to assess self-determined motivation for PE (Fitton-Davies et al., Unpublished). All children in each year 1 class will be asked to draw a picture of “what they like about PE” on one side of a piece of A4 paper and conversely “what they don’t like about PE” on the other. Due to the time necessary to administer and analyze MAT-PE, a random sub-sample of participants (∼*n* = 5 per class) will be selected to participate in 1:1 activities with a researcher. This random sample will be selected from a pool of research children, whom the class teacher will have identified as wishing to talk to researchers, and with a sufficient level of English verbal skills to be able to have a conversation with an adult. The 1:1 activities will take place in a quiet open space outside of the classroom (e.g., school library) where the researcher can be overlooked but not overheard and the conversation between the child and researcher will be recorded using a Dictaphone. The 1:1 activities will commence with an icebreaker activity to relax and build rapport between the researcher and child (a PE themed pair-matching card game). The researcher will then ask the child to describe their drawing(s) and ask questions in order to ascertain information about the picture stimulated from its content. This will be followed by a series of activities including the use of resource cards to explore needs satisfaction during PE lessons in relation to (i) relatedness, (ii) competence, and (iii) autonomy (Ryan and Deci, 2000). The final activity will involve each child being presented with a picture that represents each level of regulation along the self-determined motivation continuum that is coupled with a stem (e.g., ‘*I do PE because it is fun’*). Each stem will be read aloud to the child and clarification given if needed. The child will then be asked to pick their favorite reasons for taking part in PE, which they are subsequently asked to rank (first being most important to them, last being least important). Each 1:1 session will last around 15–20 min. Audio recordings will be transcribed and content analysis will be conducted through the use of a codebook so as to determine changes in basic psychological need satisfaction and self-determined motivation. Thematic analysis will also be conducted so as to capture information from the children around their PE experiences which may have been impacted by the intervention.

##### Executive functions

Under the guidance of a trained member of the research team (1:1), in a quiet space outside the classroom (e.g., the library), individual children will be asked to work through three age-appropriate activities from the National Institute for Health (NIH) Toolbox (Gershon et al., 2010) to assess the three core executive functions. The NIH Toolbox is a comprehensive set of neuro-behavioral measurements that quickly assess cognitive, emotional, sensory, and motor functions from the convenience of an iPad. Each child will complete three cognitive activities lasting 15 min in total: inhibitory control is assessed through The Flanker Test (3 min), cognitive flexibility through the dimension card sort (4 min), and working memory via a list sorting task (7 min). The NIH toolbox has well-established validity and reliability for use with children aged 3–15 years (Weintraub et al., 2013).

##### Self-regulation

Children’s self-regulation will be assessed using the Strength and Difficulties Questionnaire (SDQ; Goodman, 1997; Stone et al., 2010), which will be completed by class teachers for each participating child at each time point. The SDQ is a brief behavioral screening questionnaire consisting of 25 items within five subscales (emotional, conduct, hyperactivity, peer and prosocial), and has demonstrated good reliability and validity across several studies (Essau et al., 2012). There are five items on each subscale with each item scored 0, 1, or 2. Scores therefore range from 0 to 10 for each subscale, with 10 indicating higher levels of difficulties (emotional, conduct, hyperactivity, peer subscales) or strengths (prosocial subscale) and 0 indicating lower levels. A total difficulties score is also generated by summing scores from all the scales except the prosocial scale, with scores ranging from 0 (low) to 50 (high).

Each child’s self-regulation will also be assessed by researchers using the RCS (Lakes, 2012, 2013). The RCS is an observer-rated measure of children’s responses to challenges in an obstacle course. The course is designed to vary demand and challenge and takes 10–15 min to complete in a school hall/outside school playground. The trained observer rates children on 16 items comprising bipolar adjectives (e.g., Vulnerable—Invincible), which are rated on seven-point scales (scored 1–7). Negatively worded items are reversed prior to aggregation, so that possible scores on all items ranged from 1 to 7, with higher scores indicating greater self-regulation. Items are summed to assess self-regulation within three subscales: “Cognitive” (six items, scoring range from 6 to 42), “Affective” (seven items, scoring range from 7 to 49) and “Physical/Motor” (three items, scoring range from 3 to 21).

##### Anthropometrics

Children’s height, sitting height, waist and body mass will be measured with an accuracy of 0.1 cm and 0.1 kg, respectively (Lohman et al., 1988). Height and sitting height will be assessed with a portable stadiometer (Leicester Height Measure, SECA, Birmingham, United Kingdom) and body mass will be assessed using digital scales (Tanita WB100-MA, Tanita Europe, Netherlands). Waist circumference will be measured around the navel region. Measurements will be taken without shoes and whilst wearing light clothing, taking approximately 5 min. Height and weight values will be used to examine weight status through the International Obesity Task Forces age and sex adjusted body mass index (BMI) growth-reference to enable international comparisons (Cole et al., 2000).

### Process Evaluation

Informed by the RE-AIM framework and previous literature (Glasgow et al., 1999; Linnan et al., 2002) as well as by the UK Medical Research Council guidance for process evaluation that advocates exploring context, implementation, impact and outcomes (Moore et al., 2015) a pragmatic process evaluation design will examine intervention *context*, *reach*, *dose*, *fidelity*, *acceptability implementation*, *impact*, *acceptability*, and *sustainability*.

*Reach* will be assessed using school administrative data on child demographics and school registers. Teachers (control schools) and SAMPLE-PE coaches (intervention schools) will be asked to log the number of PE lessons implemented at each school, and the duration of each PE lesson in minutes to determine. Direct observations of PE lessons by researchers and coaches’ logs will be used to examine *dose delivered*, *fidelity* and participant responsiveness (*dose received*). Specifically, in each intervention and a subsample of control schools, three lessons from each class (one in every 5-week phase of delivery) for a total of approximately 50 lessons will be audio- and video- recorded, using a wireless microphone and video camera (situated to capture the whole class and deliverer).

Video footage will subsequently be analyzed by trained researchers to assess whether the intervention was delivered as intended (*fidelity*) using developed observation checklists for Non-linear and Linear pedagogies, respectively. Intervention fidelity will be confirmed if (i) the Non-linear pedagogy intervention schools’ PE lessons show greater implementation of Non-linear pedagogical principles than Linear and control schools PE lessons, and (ii) the reverse is true for Linear pedagogy intervention schools’ PE lessons. Video recordings of PE lessons will also be retrospectively coded using established observation checklists to examine SAMPLE-PE coach (intervention schools) and teacher (control schools) behaviors in relation to promoting children’s moderate-to-vigorous physical activity (SOFIT+; Weaver et al., 2016; Fairclough et al., 2018) and supporting or thwarting children’s psychological needs for relatedness, competence, and autonomy (Smith et al., 2015). Researchers will also record the number of children participating in lessons, and the number of staff present and collect data on the themes and types of activities undertaken within the control group’s PE lessons.

*Participant responsiveness*. refers to how responsive participants are to an intervention (Durlak and Dupre, 2008). For the purposes of this process evaluation, we will examine participant responsiveness in terms of children’s self-determined motivation, psychological needs satisfaction and physical activity levels within the observed PE lessons (15 lessons at each three time points). Psychological need satisfaction and enjoyment of the PE lesson from a child perspective will be assessed at the end of each observed PE lesson. Physical activity will be assessed during each observed lesson. In terms of self-determined motivation, immediately following the lesson, all research children (those within both experimental arms and three control schools) will complete brief measures of relatedness, autonomy and competence need satisfaction on a 1:1 basis with trained researchers. For relatedness, we will look to explore the quantity of social interactions. In line with Sebanc (2003) children will be asked by a member of the research team to identify which children within their class they worked with during that lesson from a school class photo list. For competence, children will be asked *how good were you at things during that PE lesson?* This will be measured on a 1–5 star rating scale: 1 being not very good and 5 being very good. For autonomy, children will be asked *did you get to do any choosing during that PE lesson?* The answer format is on a two-layer response where they first choose either ‘yes’ or ‘no.’ Depending on their initial response, they will be asked if this is ‘sometimes yes’ or always yes,’ or ‘sometimes no,’ or ‘always no.’ For enjoyment, as children leave the PE lesson, they will be asked to tap on 1 of 3 posters situated on a wall by the exit door displaying an emoji face depicted either as boring, ok or fun. Children’s actions will be video recorded by a research assistant. To assess participant responsiveness in terms of physical activity levels, a sub-sample of children (50% of the research participants in each class) will be randomly-selected to wear an Actigraph GT9X+ accelerometer (Actigraph, Pensacola, FL, United States) on their non-dominant wrist within each PE lesson observation. The time that the teacher commences and ends the lesson will be recorded by a research assistant, and used to calculate the proportion of time children spent in moderate-to-vigorous physical activity.

A qualitative methodology, will be utilized to explore the experiences and perceptions of key stakeholders within intervention schools with regards to *context*, *fidelity*, *implementation*, *impact*, and *acceptability*, and *sustainability*. Utilizing the interpretivist paradigm, it is recognized that human action and interaction such as PE lessons, is experienced subjectively evaluated through individual meaning making (McKenzie et al., 1997). Thus, the effectiveness of an intervention, such as SAMPLE-PE, is inherently linked to the experiences and perceptions of key stakeholders such as teachers. Collecting and analyzing these perceptions, through interpretivist qualitative methods is, therefore, an essential part of a process evaluation (Cheng and Metcalfe, 2018). To that end, qualitative methods are an appropriate methodology to gather data (Smith and McGannon, 2018).

Through interviews, researchers will explore: (1) the *fidelity* of the intervention; (2) *implementation* and *impact*; and (3) *acceptability* and *sustainability* of *Linear* and Non-linear pedagogy intervention curriculums. The sample is purposive in that individuals with the experience of intervention will be recruited. It is also iterative, because as the intervention proceeds, the sample size may increase to include other stakeholders, e.g., teaching assistants. Importantly, the process evaluation not only gathers the experiences and perceptions of stakeholders such as teachers, but a process evaluation can also describe the context in which interventions were experienced. This will be captured through structured interviews with head teachers of intervention schools who are well-placed to describe the school as a whole. These interviews will explore school policy, funding, support, equipment, time allocation for PE, and potential for scale-up of the interventions, as well as any other aspects of the complex school environment that may have influenced the intervention and outcomes.

To collect interview data, a combination of skype, face-to-face and email interviews will be utilized. More specifically, participants will be offered the opportunity to share their experiences and perceptions in the format that best enables them to do so. This choice enables participants to exercise their autonomy (Orb et al., 2001). Structured interview schedules have been developed (Supplementary File 3) in order to focus attention on the context, fidelity, implementation, impact, acceptability and sustainability of the intervention across both *Linear* and Non-linear Pedagogy schools. The use of a structured interview schedule will ensure that interviews will be conducted in a consistent manner regardless of medium, e.g., face-to-face or email. The structured format of the interview schedule will also ensure that any researcher bias is ‘managed’ in order to maintain equipoise as far as possible (Eborall et al., 2014). Interviews will be transcribed and analyzed using thematic analysis (Clarke and Braun, 2013). To ensure rigor during the data collection and analysis processes, co-researchers will act as critical friends (Smith and McGannon, 2018). This will involve reviewing the structured interview schedule to identify leading questions, and reviewing coding and themes to ensure verisimilitude with the data.

### Data Analysis

Linear-mixed models will be conducted to examine the effects of the SAMPLE-PE intervention on the main outcomes of the study (i.e., movement competence development) in the short-term (post-intervention) and medium-term (at follow-up). Separate analyses will be conducted for each outcome measure. Mixed models are used to account for the nested structure of the data. The significance level will be set *p* < 0.05 for all statistical analyses. Regression coefficients for the group variables (with a “0” and “1” dummy coding) will reflect average differences in the outcome variables over time. Potential effects of confounding factors such as sex, age, ethnicity, and deprivation will be examined in the hierarchical linear regression analyses. Mediation analyses will be conducted to examine hypothesized mediating pathways that might explain the expected intervention effects in non-motor (cognitive and affective) domains through gains in actual (Pesce et al., 2016a; Sánchez-López et al., 2019) and perceived movement competence. Attrition analyses comparing children who completed the study and those who dropped out will also be performed. Analyses will be conducted using R and follow an intention-to-treat approach.

## Discussion

The SAMPLE-PE study aims to examine the efficacy of two different pedagogical approaches to PE (Linear or Non-linear) upon children living in deprived areas. Each approach is informed by movement learning theories used to support the design of learning experiences which, beyond mere movement learning outcomes, are also tailored to support the development of non-motor (cognitive affective) aspects of children’s physical literacy journey. In this frame of expected outcomes in physical and wider domains, the study also aims at providing important insights into the inter-connected nature of physical, affective and cognitive developments that can be elicited by SAMPLE-PE. To deliver these pedagogical models effectively, the coaches will need to possess an in-depth knowledge of the respective pedagogy and learning design principles to improve movement competence. Coaches will receive a comprehensive and extensive training programme from the research team to enable them to deliver the SAMPLE-PE intervention curriculums. A potential limitation to the evaluation is that we do not have the capacity to examine the fidelity of the training, though we will measure the coaches’ ability to deliver the interventions in accordance with the corresponding pedagogy via direct observation of a sub-sample of PE lessons.

The findings of this study should further develop pedagogical practice, inform learning design within PE, shed new light on how to enhance children’s development of movement competence and, more broadly, lead to a better understanding of how to foster physical literacy in the children who need it most. As such, the study could have significant implications for the primary school PE curriculum and for career professional development and training offered to sports coaches and specialist/generalist primary school teachers. Furthermore, the comprehensive mixed methods process evaluation and use of robust outcome measures should provide novel, inter-disciplinary insight into movement competence as a driver of perceived competence, motivation, cognition and physical activity, and extend current knowledge about the effectiveness of PE interventions. The study has therefore the potential to raise standards and the value of PE, and progress to a scaled-up, effectiveness trial involving classroom teachers in the future.

## Author Contributions

JR and LF conceived the study. All authors were involved in the design of the study protocol and assisted with the drafting and revising of the manuscript, read and approved the final manuscript.

## Conflict of Interest

The authors declare that the research was conducted in the absence of any commercial or financial relationships that could be construed as a potential conflict of interest.

## Abbreviations

EUPEA: European Physical Education Association
NIH: National Institutes for Health
OECD: Organisation for Economic Co-operation and Development
PE: Physical Education
RCS: Response to Challenge Scale
RCT: Randomized Controlled Trial
SAMPLE-PE: Skill Acquisition Methods fostering Physical Literacy in Early – Physical Education
SDQ: Strengths and Difficulties Questionnaire
STEP, Space: Task, Equipment People
TGMD-3: Test of Gross Motor Development Third Edition
UNESCO, United Nations Educational: Scientific and Cultural Organization.
