# Supplementary material for: Skill Acquisition Methods Fostering Physical Literacy in Early-Physical Education (SAMPLE-PE): Rationale and Study Protocol for a Cluster Randomized Controlled Trial in 5–6-Year-Old Children From Deprived Areas of North West England
Source: Front Psychol. 2020 Jun 17;11:1228. doi: 10.3389/fpsyg.2020.01228 (PMC7311787; doi:10.3389/fpsyg.2020.01228)
Supplement: Supplementary file 1 [file Table_1.docx]

**GYMNASTIC LINEAR LESSON EXAMPLE**

| **Lesson’s outcome** | | Children will be able to demonstrate mastery in a the log roll and in rocking back and forward and will be able to use these skills in a gymnastic performance | |
| --- | --- | --- | --- |
| **Linear transitions using Gentile’s taxonomy** | | **Body:** from no body transport to body transport  **Object:** from no object manipulation to object manipulation  **Motion:** from environment still to environment moving  **Inter-trial Variability:** from no inter-trial variability to inter-trial variability | |
| **Activity** | **Time** | **Activity Description** | **Diagram of basic set-up** |
| **Warm up** | **5min** | In this lesson children play foxes and spiders where the aim of the game is for the foxes to catch the spiders. If the spiders are caught, they freeze in to a balance position they learnt during the previous lesson.  *The warm up activity is used to revisit the skills learnt in the previous lesson. Skills are practiced in a game environment (challenging end of the Gentiles Taxonomy).* | Fox  Mat  Spider   \|  \| \| --- \| |
| **Activity 1: Log Roll** | **10min** | *Begins lesson at the bottom of Gentiles Taxonomy (progresses through taxonomy 1, 2, 3 are examples of this)*   1. The coach explains and demonstrates the starting position of the log roll: laying down on a mat with legs straight while keeping arms straight above the head. Each child practices the starting position while the coach moves around the class giving corrective feedback. 2. When the coach is happy that a child can master the basic shape of the log roll he/she invites the child to start practicing rolling across the mat. 3. The coach can introduce a progression of difficulty by proposing different arms positions and different equipment for the children to practice the roll on or by asking the children to synchronise the roll with a partner. | \|  \| \| --- \|     Child  Coach  Mat |
| **Activity 2: The Rock (Teaching progression for the forward roll)** | **15min** | 1. The coach explains and demonstrates the starting position: sitting down on a mat maintaining the hold of the legs pulled in tight to the chest. Children practice getting in and out of the position. 2. The coach explains and demonstrates rocking backwards until the base the neck touches the mat and then rocking forward while keeping the body tight in the starting position. Children practice the skill until they demonstrate mastery. 3. Children practice with a partner trying to synchronise their rocking so they stand up together.   *Throughout both of these activities the coach uses challenge point framework to support learning. The coach also provides a demonstration accompanied by verbal cues.* | \|  \| \| --- \|     Child  Coach  Mat |
| **Final Activity: The Performance** | **15min** | The coach asks children to create a routine on the mat that must include the two rolls learnt during the lesson and a motor skill learnt in previous weeks.   1. Children create their own routine. Based on skills learnt and high MC will integrate previously learnt skills. 2. The coach divides the class in pairs and children combine their routine. 3. Each pair of children show the routine to the class.   *The coach uses challenge point framework to support learning in the children’s final performance.* | \|  \| \| --- \|     Direction  Child  Coach  Mat |
| **Cool down** | **5min** | The coach asks to walk around the hall and progressively slow down the walking pace until stopping and sitting down on a mat, then the coach invites children to copy the mobility exercises he/she performs. The coach asks questions about what children experienced and what they learnt during the lesson.  *The coach uses the cool down to verify whether children retained the information presented during the lesson about log roll and rocking.* | \|  \| \| --- \|     Child  Coach  Mat |
