# Supplementary figures and images for: Skill Acquisition Methods Fostering Physical Literacy in Early-Physical Education (SAMPLE-PE): Rationale and Study Protocol for a Cluster Randomized Controlled Trial in 5–6-Year-Old Children From Deprived Areas of North West England
Source: Front Psychol. 2020 Jun 17;11:1228. doi: 10.3389/fpsyg.2020.01228 (PMC7311787; doi:10.3389/fpsyg.2020.01228)

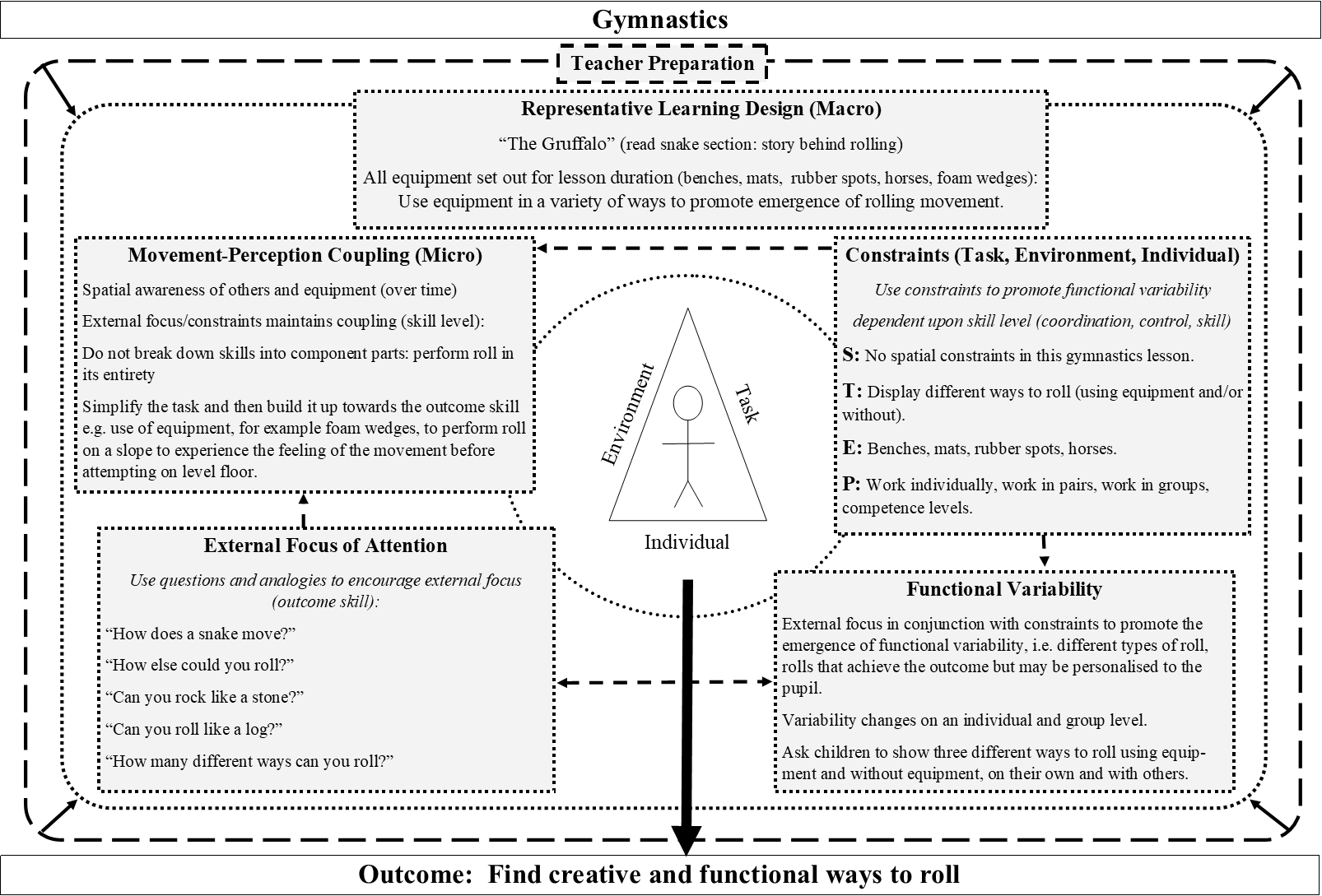

Supplement: Supplementary file 2 [file Table_2.docx]
