# Supplementary material for: Skill Acquisition Methods Fostering Physical Literacy in Early-Physical Education (SAMPLE-PE): Rationale and Study Protocol for a Cluster Randomized Controlled Trial in 5–6-Year-Old Children From Deprived Areas of North West England
Source: Front Psychol. 2020 Jun 17;11:1228. doi: 10.3389/fpsyg.2020.01228 (PMC7311787; doi:10.3389/fpsyg.2020.01228)
Supplement: Supplementary file 3 [file Table_3.docx]

**World Health Organization Trial Registration Data Set.**

| **Data Category** | **Information** |
| --- | --- |
| Primary registry and trial identifying number | ClinicalTrials.gov  NCT03551366 |
| Date of registration in primary registry | 6^th^ September, 2018 |
| Secondary identifying numbers | n/a |
| Source(s) of monetary or material support | Liverpool John Moores University |
| Primary sponsor | Liverpool John Moores University |
| Secondary sponsor(s) | N/A |
| Contact for public queries | J.R.Rudd@ljmu.ac.uk |
| Contact for scientific queries | j.r.rudd@ljmu.ac.uk |
| Public title | Influence of Different Physical Education Pedagogical Approaches on the Health and Development of 5-6 Year Old children |
| Scientific title | Skill Acquisition Methods fostering Physical Literacy in Early-Physical Education (SAMPLE-PE) in 5-6 year old children: Rationale and study protocol for a cluster randomised controlled trial |
| Countries of recruitment | England |
| Health condition(s) or problem(s) studied | Child Development  Health Behaviour  Physical Activity |
| Intervention(s) | Behavioural: Linear  Behavioural: Nonlinear |
| Key inclusion and exclusion criteria | Ages eligible: Children aged 5-6 years  Sexes eligible: Both  Exclusion criteria: children diagnosed with health or coordination issues that could affect motor competency |
| Study type | Intervention (Clinical Trial) |
| Date of first enrolment | November 2017 |
| Target sample size | 314 |
| Recruitment status | Active, not recruiting |
| Primary outcome(s) | Motor competence |
| Key secondary outcomes | Physical activity  Perceived motor competence  Motivation  Executive functions  Self-regulation |
